# The Crosstalk Between Endothelial Cells, Smooth Muscle Cells, and Macrophages in Atherosclerosis

**DOI:** 10.3390/ijms26041457

**Published:** 2025-02-10

**Authors:** Sihe Gong, Yanni Li, Kaijie Yan, Zhonghong Shi, Jing Leng, Yimin Bao, Ke Ning

**Affiliations:** 1 School of Integrative Medicine, Shanghai University of Traditional Chinese Medicine, No. 1200 Cailun Road, Shanghai 201203, China; gsh15536576127@163.com (S.G.); annie0314@163.com (Y.L.); ykj0917@126.com (K.Y.); instable_benzene@hotmail.com (Z.S.); 2 School of Traditional Chinese Medicine, Shanghai University of Traditional Chinese Medicine, No. 1200 Cailun Road, Shanghai 201203, China; 3 Preclinical Department, Shanghai Municipal Hospital of Traditional Chinese Medicine, Shanghai University of Traditional Chinese Medicine, Shanghai 200071, China; lengjing2022szy@163.com

**Keywords:** atherosclerosis, endothelial cells, smooth muscle cells, macrophages

## Abstract

Atherosclerosis (AS) is a chronic inflammatory vascular disease closely tied to cellular metabolism. Recent genome-wide association study data have suggested the significant roles of endothelial cells, smooth muscle cells, and macrophages in the regression and exacerbation of AS. However, the impact of cellular crosstalk and cellular metabolic derangements on disease progression in AS is vaguely understood. In this review, we analyze the roles of the three cell types in AS. We also summarize the crosstalk between the two of them, and the associated molecules and consequences involved. In addition, we emphasize potential therapeutic targets and highlight the importance of the three-cell co-culture model and extracellular vesicles in AS-related research, providing ideas for future studies.

## 1. Introduction

With the improvement of people’s living standard and concern for life and health, cardiovascular diseases have been increasingly emphasized. Atherosclerosis (AS), as an important cardiovascular disease, is a chronic inflammatory vascular disease that usually occurs in large- and medium-sized arteries, and even leads to cardiovascular diseases such as coronary heart disease, cerebral infarction, and peripheral vascular disease in severe cases [1]. Various reasons such as heredity, poor dietary habits, chronic diseases, etc. can cause AS, which produces stimulation to human cells. The exchange of information between cells causes changes in cellular physiological activity, which in turn alters the body’s integrated response to the external environment and the process of growth and development [2,3]. Therefore, it is of great significance to study the pathogenesis of AS and to explore the mechanism of intercellular interaction for the treatment of cardiovascular diseases.

The traditional theory of lipid infiltration believes that elevated plasma lipid levels promote the influx and local deposition of substantial lipids, particularly cholesterol, into the arterial wall. Upon oxidation, the deposited lipids elicit the accumulation and phagocytosis by macrophages and smooth muscle cells (SMCs), leading to the formation of foam cells and subsequent thickening of the vascular intima [4], ultimately culminating in the development of atherosclerotic lesions.

**Figure 1:**
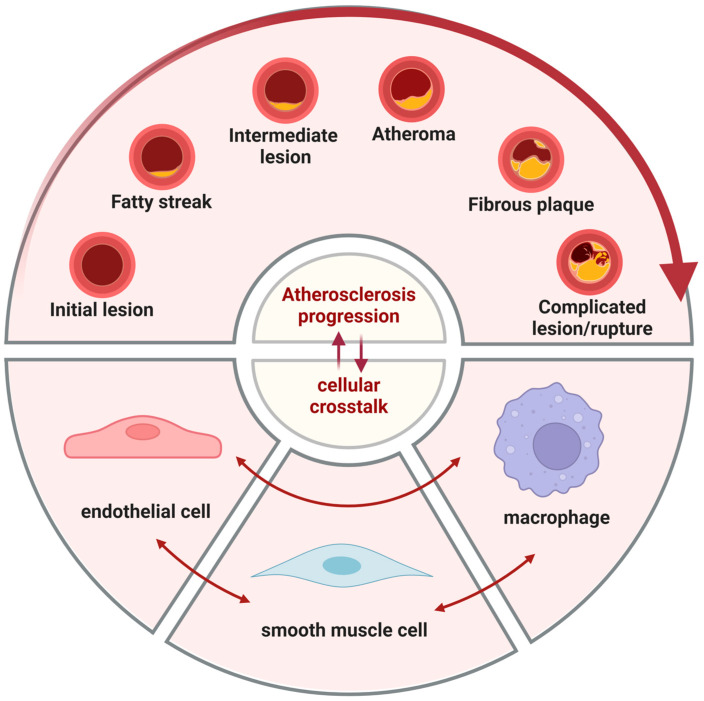
Intercellular communication affects the progression of atherosclerosis. In atherosclerosis, initial changes at the cellular and molecular levels lead to endothelial injury, which may not immediately cause luminal narrowing or impairment of tissues and organs. The foam cells then gather below the endothelium cells and appear as yellow fat splotches. This is followed by the accumulation of lipids within and outside the cells, generating lipid pools that disrupt the intimal structure and deform the arterial wall. Smooth muscle cells migrate to participate in the formation of a fibrous cap, leading to the emergence of white plaques that protrude into the arterial lumen, causing narrowing and the formation of atherosclerotic and fibrous plaques. On the basis of these plaques, secondary lesions such as hemorrhage, necrosis, ulceration, calcification, and mural thrombosis can occur, potentially triggering acute cardiovascular events. In this process, endothelial cells, smooth muscle cells, and macrophages all play important roles.

Russell Ross proposed the doctrine of endothelial damage and inflammation in 1999 [5]. He clarified that the development of AS is accompanied by the involvement of various inflammatory cells and inflammatory factors, among which the damage of arterial endothelium is the main reason for the development of AS. AS initiates with endothelial dysfunction induced by perturbed shear stress. Mechanical forces exerted on the endothelium, such as increased shear stress, circumferential stretch, or luminal hypertension, can modulate gene expression, structure, and function, thereby inducing alterations in biochemical pathways. This process is termed “mechanotransduction” [6]. It leads to increased permeability of endothelial cells (ECs) to circulating low-density lipoprotein (LDL) [7], resulting in a substantial deposition of lipids within the damaged intima, which are subsequently oxidized into oxidized low-density lipoprotein (ox-LDL). Upon stimulation, such as by dyslipidemia, hypertension, or pro-inflammatory mediators, ECs express adhesion molecules, cytokines, and chemokines [8], including MCP-1, intercellular adhesion molecule (ICAM) -1, vascular cell adhesion molecule (VCAM)-1, E-selectin, and P-selectin, which attract monocytes to the damaged area and induce their migration into the subendothelial space. Activated ECs also express macrophage colony-stimulating factor, aiding in the transformation of infiltrating monocytes into macrophages. Macrophages, through the expression of pattern recognition receptors such as scavenger receptors, engulf large amounts of ox-LDL, leading to the formation of foam cells [9]. These foam cells aggregate to form lipid streaks, which represent early atherosclerotic lesions. Concurrently, SMCs proliferate and migrate, and even transform into foam cells, contributing to the formation of atherosclerotic plaques [10]. As plaques develop, the accumulation of lipids and necrotic debris within them increases, gradually compromising plaque stability [11]. Under certain conditions, such as elevated blood pressure or hemodynamic stress, plaques may rupture, triggering hemorrhage and thrombosis [12,13] (Figure 1). This shows that ECs, SMCs, and macrophages play an important role in the formation of AS. According to a research using public genome-wide association study data [14], genes for cardiovascular disease susceptibility were determined to be enriched in diseased macrophages, ECs, and SMCs, such as SHE, AMPD2, and ITGB [14]. This undoubtedly shows the importance of these three cells in cardiovascular disease.

In this review, we discuss the roles of ECs, SMCs, and macrophages in AS based on the latest research advances, and summarize the signaling communication process between the three cell types two by two for further research and exploration.

## 2. Roles of Endothelial Cells, Smooth Muscle Cells, and Macrophages in Atherosclerosis

### 2.1. Endothelial Cells

It is commonly known that ECs are mainly involved in constituting the inner wall of arterial vessels and participate in the process of selective fluid osmotic exchange between blood and tissue fluids. Meanwhile, their intercellular junctions and transmembrane transport of vesicles contribute to the transport of substances and exchange of information in blood and tissue cells [15]. Normally, ECs in the human vasculature are in a relatively stable state, but in pathological states like injury or hypoxia, the ECs can be activated rapidly and undergo neovascularization that provides oxygen and nutrients to the tissues in order to slow down further damage [16]. However, the continuous activation of the EC can lead to a decompensated phase, resulting in endothelial dysfunction and AS.

### 2.2. Smooth Muscle Cells

One of the key features of AS is the proliferation and migration of SMCs, which are found in the tunica media [17,18]. SMCs are highly plastic and can undergo phenotypic transformation through signaling pathways such as Wnt, Notch, and transforming growth factor (TGF)-β [19,20,21]. In normal individuals, the SMCs in the arterial vessel wall mostly exhibit a contractile phenotype, which have limited proliferative and migratory potential, and serve to maintain vascular elasticity and constrict blood vessels [22]. However, SMCs in pathological conditions transform into a synthetic phenotype with enhanced capabilities for cell migration and proliferation, which can be specifically classified into proliferative–migratory, inflammatory, and ossification/osteogenic phenotypes, etc. [22]. This phenotype is able to synthesize a large amount of extracellular matrix (ECM), collagen, and bone-bridging proteins, among other things. Currently, some studies suggest that in the early stage of AS, abnormally proliferating SMCs can promote plaque formation, whereas in the late stage, SMCs have the ability to prevent the rupture of the fibrous cap to stabilize the plaque [18]. Thus, it is clear that SMCs play different roles at different periods of AS.

### 2.3. Macrophages

Macrophages are mainly differentiated from different kinds of cells, such as monocytes, ECs, and SMCs, and can also be proliferated from macrophages in local tissues. They can generally be divided into three groups: recruited macrophages, tissue-resident macrophages, and perivascular macrophages [23]. These cells become activated when they are stimulated, which results in more active cell metabolism, stronger phagocytosis, etc. [24]. There are two main phenotypes of macrophages: M1 and M2. It is now believed that the M1 phenotype stimulates the production of pro-inflammatory factors like tumor necrosis factor (TNF) -α, interleukin (IL)-1β, and nitric oxide (NO), which play a critical part in the initial process of AS [25]. On the other hand, the M2 phenotype secretes pro-angiogenic anti-inflammatory cytokines like TGF-β and IL-10, which support tissue repair and angiogenesis [25]. In addition to the two phenotypes, M1 and M2, cellular phenotypes such as M(Hb), Mhem, HA-mac, Mox, and M4 [26,27] have different characteristics and are all important in AS.

## 3. Crosstalk

Crosstalk between the three cell types can be broadly classified into three categories: direct cell-to-cell communication, paracrine secretion of vasoactive substances and extracellular vesicles (EVs), and cell–matrix interactions.

### 3.1. Crosstalk Between Endothelial Cells and Smooth Muscle Cells

**Figure 2:**
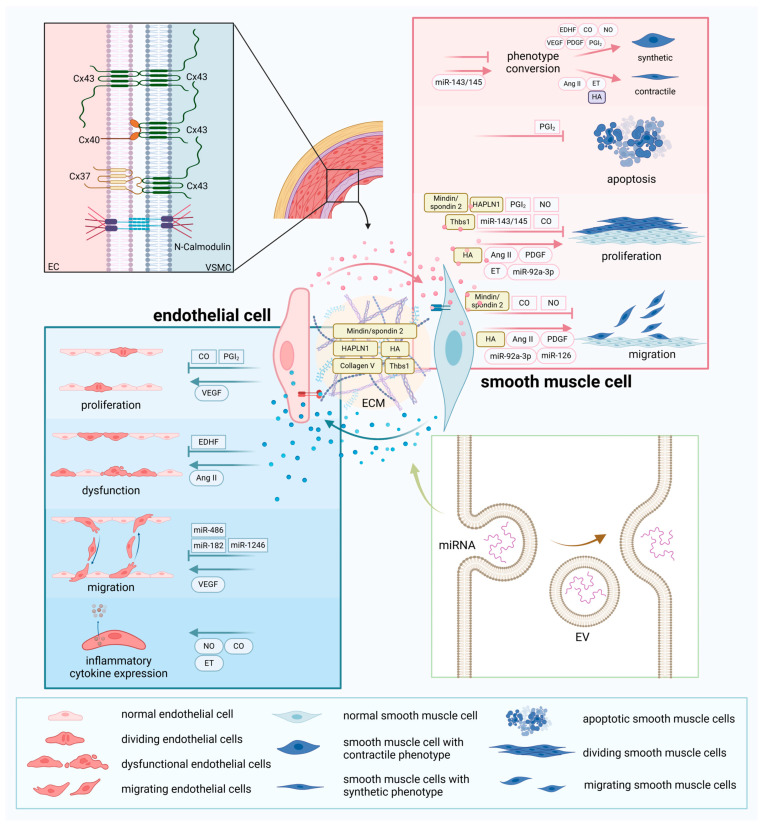
Crosstalk between endothelial cells and smooth muscle cells and the extracellular vesicle transfer process. Endothelial cells (ECs) and smooth muscle cells (SMCs) communicate directly through gap junctions and adhesion junctions, involving proteins such as Connexin (Cx) 37, Cx40, Cx43, and N-cadherin. The two cells can also influence each other through various vasoactive substances and extracellular vesicles. Extracellular vesicles are formed from the plasma membrane and transport molecules such as miRNAs and peptides. These chemicals regulate EC proliferation, dysfunction, migration, and expression of inflammatory factors. They can also alter SMC phenotypic transition, apoptosis, proliferation, migration, and expression of inflammatory factors. Furthermore, the extracellular matrix can mediate communication between ECs and SMCs through its protein or carbohydrate components and mechanical transmission.

The crosstalk between ECs and SMCs exerts a dual impact. On one hand, ECs modulate the phenotypic switching, apoptosis, proliferation, and migration of SMCs. On the other hand, SMCs influence the proliferation, dysfunction, migration, and expression of inflammatory cytokines in ECs (Figure 2).

#### 3.1.1. Direct Contact Between Endothelial Cells and Smooth Muscle Cells

The direct communication process between ECs and SMCs that are in contact with each other can occur through gap junctions and adhesion junctions [28,29].

In gap junctions, six identical connexin proteins form a connexon, and the connexons on the surfaces of adjacent cells connect to form channels through which signaling molecules can pass. This allows metabolic coupling between cells, playing a crucial role in cell growth and proliferation [30]. The gap junctions that have been investigated between EC and SMC are primarily myoendothelial gap junctions (MEGJs), which are located at the interface of adjacent protrusions formed by ECs and SMCs, and involved in the regulation of vascular tension [31]. The major connexins (Cx) in the vascular system include Cx37, Cx40, Cx43, and Cx45. All connexins except Cx45 are present in the MEGJs between ECs and SMCs [28]. Since ECs predominantly express Cx37, Cx40, and Cx43, and SMCs mainly express Cx43 and Cx45 [28], the MEGJ between them is primarily composed of Cx43/Cx43, Cx40/Cx43, and Cx37/Cx43, with the specific type also related to individual species and vascular types. In the ECs covering late-stage atherosclerotic plaques, the expression of Cx40 and Cx37 is undetectable. However, these connexins are highly expressed in the endothelium around the plaque and during the development of AS [32]. Blocking the expression of Cx43 induced by ox-LDL can activate SMCs autophagy by inhibiting the PI3K/AKT/mTOR signaling pathway, thereby inhibiting SMC hyperproliferation and migration [33], as well as foam cell formation [34]. Additionally, Cx37 in injured arteries can promote gap junction communication by phosphorylating Cx43 via the Akt pathway, which in turn limits SMC proliferation and intimal hyperplasia [35,36]. Gap junctions containing Cx43 can also mediate signal transmission from SMCs to ECs to regulate vascular tone. Inositol 1,4,5-trisphosphate (IP_3_) originating from SMCs can traverse via MEGJ to reach ECs, where it binds to endogenous IP_3_ receptors, leading to an elevation of intracellular Ca^2+^ levels in ECs. Elevated Ca^2+^ levels in SMCs stimulate the generation of IP_3_, and blockade of IP_3_ receptors can prevent the subsequent increase in Ca^2+^ levels within ECs [37]. Furthermore, the function of connexins is regulated by cytokines; for instance, in vitro, bacterial lipopolysaccharide (LPS), TNF-α, and IL-1β can selectively inhibit intercellular communication between human vascular gap junction cells [38]. The dynamic alterations in connexin expression during the progression of AS and their subsequent impact on disease trajectory remain to be fully elucidated.

In addition to gap junctions, ECs and SMCs are also connected by adhesion junctions. N-cadherin, an adhesion molecule that can mediate the connection between cells [39], is present in the elastic membrane between the EC and SMC layers, suggesting that N-cadherin may mediate EC–SMC interactions [29]. New studies have shown that inhibiting N-cadherin function can inhibit SMC migration and promote EC survival, thereby delaying endothelial thickening [40,41]. Additionally, VE-cadherin participates in the adhesion junctions between ECs, but EC proliferation induced by mechanical stretch necessitates contact with SMCs, whereas SMC proliferation occurs without the need for interaction with other cells [42]. This highlights the significance of direct cell-to-cell communication between the two cell types for AS development.

#### 3.1.2. Paracrine Secretion Between Endothelial Cells and Smooth Muscle Cells

##### Vasoactive Substance Between Endothelial Cells and Smooth Muscle Cells

Endothelium-derived diastolic factors such as NO, carbon monoxide (CO), prostacyclin (PGI_2_), and endothelium-derived hyperpolarizing factor (EDHF) lead to SMC and vasodilatory relaxation [43]. Conversely, angiotensin II (Ang II), and endothelin (ET), are endothelium-derived contracting factors that promote SMC and vasoconstriction [43]. These factors can also be secreted by SMCs and consequently influence the growth and metabolic processes of ECs. Moreover, growth factors such as platelet-derived growth factor (PDGF) [44] and vascular endothelial growth factor (VEGF) [45] also regulate cell activity.

Endothelial nitric oxide synthase (eNOS) can oxidize L-arginine to promote the production of NO after external or physiological stimulation [46]. The newly formed NO diffuses rapidly to the cell membrane of ECs, thereby changing the concentration of cyclic guanosine monophosphate (cGMP) [47], which causes vasodilation in SMCs [48,49], and reversibly inhibits SMCs migration [50] and proliferation [51]. This process also promotes the transformation of SMCs from a contractile to a synthetic phenotype through the soluble guanylyl cyclase (sGC)-PRKG1 signaling pathway [52]. Moreover, recent studies have shown that eNOS can also be expressed in SMCs [53], and it has been demonstrated that the interaction of eNOS with cell adhesion molecules (CAMs) in SMCs is promoted by ECs. This suggests that in the process of new vessel formation, SMCs may compensate for the production of NO by ECs to regulate vascular tone.

CO can be produced by heme oxygenase catalysis [54]. CO shares many similarities with NO, as it can also bind to and activate sGC, leading to an increase in cGMP levels in cells and subsequently causing vasodilation of SMCs. In addition to these, CO can inhibit SMC proliferation and migration. For example, CO inhibits SMC proliferation and neointimal formation by activating p38 mitogen-activated protein kinase (MAPK) and p21 (Waf1/Cip1) [55,56] or through the TGF-β1 pathway [57]. It also inhibits SMC migration stimulated by PDGF by reducing the enzyme activity of Nox1 [58]. On the other hand, CO released by SMCs can increase endothelial cGMP levels and reduce the expression of mitogens, ET-1, and PDGF-B under hypoxia, which in turn inhibits SMC proliferation [59]. CO can also restore TNF-α–induced downregulation of eNOS and endothelial dysfunction by inhibiting NF-κB [60]. However, the specific role of CO in AS needs further research.

PGI_2_ is a class of fatty acids produced by the metabolism of arachidonic acid via the cyclooxygenase (COX) pathway. In response to proinflammatory mediators and mechanical stress, PGI_2_ produced by ECs can bind to SMC surface receptors, activate adenylate cyclase, and generate cAMP. This subsequently activates protein kinase A, inducing relaxation of both blood vessels and SMCs, and inhibiting platelet aggregation [61,62]. PGI_2_ also can reduce SMC apoptosis and maintain the contractile phenotype through PPARα and 14-3-3 signaling pathways [63,64]. Studies have also shown that PGI_2_ receptor deficiency or inhibition of PGI_2_ production by inhibiting COX-2 can accelerate AS and thrombosis in mice [65]. Therefore, severe endothelial injury leads to insufficient PGI_2_ production, which results in SMC apoptosis, proliferation, and intimal hyperplasia. Moreover, PGI_2_ promotes the release of NO from ECs [66] while NO can also enhance the effects of PGI_2_ on SMCs.

EDHF is a class of active substances that dilate blood vessels, distinct from NO and PGI_2_. It does not refer to a specific substance but includes cytochrome P450 metabolites, hydrogen peroxide, cyclic adenosine monophosphate, and C-type natriuretic peptide, among others [67]. EDHF may serve as a compensatory pathway for NO-dependent endothelial vascular dilation, promoting vascular relaxation before the development of AS in the aorta [68,69]. Similarly, in elderly obese mice on a long-term high-fat diet, a reduction in EDHF-mediated vascular relaxation has been found. This reduction may potentially contribute to endothelial dysfunction in small resistance arteries [70,71]. In addition, the absence of the Cx40 gene slows down the EDHF-mediated spread of hyperpolarization from ECs to SMCs [72]. Therefore, EDHF also plays a role in vascular relaxation through gap junctions.

Ang II is a peptide with potent vasoconstrictive properties that is typically converted from angiotensinogen by renin and angiotensin-converting enzyme (ACE), or produced via a non-ACE-dependent system [73]. Ang II can be recognized by two different G protein-coupled receptors, the Ang II type-1 receptor (AT1R) and the Ang II type-2 receptor (AT2R). AT1R usually mediates vasoconstriction and inflammatory responses, while AT2R is generally associated with vasodilation and apoptosis [74]. Ang II can advance the progression of AS by affecting ECs through AT1R or by mediating oxidative stress [74]. For instance, Ang II, when bound to AT1R, activates the JNK pathway, which promotes the expression of ACE in ECs. Ang II-mediated oxidative stress can lead to dysregulation of NO production in ECs, resulting in endothelial dysfunction [75]. Additionally, Ang II can promote the transition of SMCs from a contractile to a synthetic phenotype, proliferation, migration, and alterations in ECM components through AT1R [75]. It can also facilitate the transformation of SMCs into foam cells by increasing the expression of the lipoprotein receptor LRP1 [76]. Moreover, AT2R inhibits the lectin-like oxidized low-density lipoprotein receptor LOX-1, and the expression of inflammatory mediators reduced in plaques from AS-simulated mice with overexpression of AT2R [77], indicating a certain anti-inflammatory effect of AT2R. Although both ECs and SMCs can produce Ang II, most studies focus on the individual effects of Ang II on ECs or SMCs, with fewer investigations into their communication through Ang II. In vitro experiments suggest that endothelial progenitor cells may inhibit Ang II–induced proliferation of vascular SMCs by blocking MAPKs and NF-κB signaling pathways and the expression of related factors, with early endothelial progenitor cells showing a more significant inhibitory effect compared to human umbilical vein ECs [78]. In summary, the crosstalk between ECs and SMCs mediated by Ang II and the role of AT2R in AS deserve further research.

ET is produced from big endothelin-1 through the action of peptidases and endothelin-converting enzymes [79]. One of its isoforms, ET-1, plays a significant role in regulating cardiovascular function. Both ECs and SMCs can secrete ET-1, which binds to two types of ET receptors, ETA and ETB, present in the human body to exert its effects. ETA receptors are primarily found on SMCs and mediate cell proliferation and vasoconstriction [80]. When ET-1 binds to ETA, it activates phospholipase C and promotes the opening of Ca^2+^ channels, leading to SMC contraction. Additionally, ET-1 activates the transcription of related genes through a cascade amplification signaling system, promoting cell proliferation and differentiation [81]. ETB receptors are mainly present on ECs and mediate vasodilation by promoting NO release [82]. ETB can utilize cytoskeletal rearrangement and Rho kinase to increase the expression of CHSY-1 in aortic ECs through the transduction of TGF-β type-I receptors [83].

PDGF is a bioactive peptide growth factor [84] that can be produced by ECs and SMCs. PDGF-B subtype was predominantly present in ECs and PDGF-A in SMCs. Influenced by some pro-inflammatory factors [85,86] and low shear stress [87], the expression of PDGF-B in ECs increases and induces the proliferation, migration, and phenotypic switching of SMCs. Similarly, decreased PDGF-B expression in ECs prevents SMC migration and proliferation, which lessens the damage-induced neointimal hyperplasia [44]. Although PDGF-A is often used as an indicator for AS development, research on its role in cell communication is relatively limited.

VEGF is a dimeric cationic glycoprotein whose family members comprises eight isoforms, including VEGF-A and VEGF-B [88,89]. These proteins can bind to vascular endothelial growth factor receptors (VEGFRs) to exert their effects. Inducing angiogenesis and increasing vascular permeability, VEGF-A plays a critical function in the proliferation and migration of tissue vascular ECs [45]. It can also upregulate endothelial eNOS through VEGFR-2/KDR, facilitating NO release and vascular relaxation [90]. Furthermore, research has demonstrated that PDGF can stimulate EC proliferation in vitro by upregulating VEGF expression in SMCs via the ERK-1/2 and AP-1 signaling pathways [91]. It can be seen that different signaling pathways can influence each other, suggesting that blocking the ERK-1/2 and AP-1 signaling pathways may provide a potential approach for treating AS.

##### Extracellular Vesicle Between Endothelial Cells and Smooth Muscle Cells

EVs are closed membrane vesicles formed by a phospholipid bilayer [92]. They serve as carriers of proteins, lipids, DNA, and mRNA, representing a novel pathway for intercellular communication [93]. EVs can be classified into three types: exosomes, microvesicles, and apoptotic bodies [94,95]. Exosomes typically have a diameter of 30–100 nm and are produced through the endosomal pathway, collecting intraluminal vesicles in multivesicular bodies, which then fuse with the plasma membrane to release exosomes [92,96]. Microvesicles have a diameter of 100–1000 nm and are formed by budding from the plasma membrane. Apoptotic bodies, with a diameter of approximately 50–5000 nm, are formed by the formation of bubbles in the plasma membrane of apoptotic cells [97]. The production and release of EVs depend on external stimuli and changes in the body’s internal environment, such as environmental stress and calcium concentration, which can alter the physiological activities of target cells [95]. ECs, SMCs, and macrophages can all produce EVs.

As one of the key molecules transported by EVs, miRNAs are short single-stranded RNA molecules composed of 18–22 nucleotides [98]. Most miRNAs in circulating plasma are found in plasma exosomes and microvesicles [99]. The miRNAs in exosomes are involved in a range of physiological and pathological processes, including AS. They play a crucial role in the communication between ECs and SMCs and in cell growth and metabolism in AS [100], such as miRNA-126, miRNA-214, miRNA-143/145, miR-204-5p, miR-92a-3p, miRNA-29a, miRNA-1246, miRNA-182, and miRNA-486, among others [101]. Under the effect of laminar shear stress in AS lesions, EC-released miR-126 can stimulate SMC migration [102]. ECs can also secrete exosomes rich in miR-214, which mediates an anti-angiogenic effect by downregulating the protein Quaking and inhibiting the expression of pro-angiogenic growth factors [103]. However, it has also been shown that miR-214 stimulates AS by inhibiting the expression of mutated ataxia telangiectasia mutated in neighboring target cells [104]. The opposite effects may be due to differences in experimental materials or mediating pathways. miR-143/145 can promote SMC differentiation and inhibit their proliferation [105]. According to studies, the shear response transcription factor Krüppel-like factor 2 stimulates ECs to secrete EVs containing miRNA-143/145 to SMCs, which inhibits the expression of miR-143/145 target genes and dedifferentiation-related genes to reduce the formation of AS lesions [100]. Activation of endothelial autophagy facilitates the transfer of miR-204-5p from ECs to SMCs via exosomes, which not only prevents EC apoptosis but also alleviates SMC calcification [106]. Exosomes from ECs can transfer miR-92a-3p to SMCs, inducing cell proliferation and migration and exacerbating inflammatory responses [107]. On the other hand, in human aortic SMCs overexpressing Krüppel-like factor 5, particularly following treatment with oxLDL, the levels of miR-29a transferred to ECs are significantly reduced. This observation suggests that both KLF5 and oxLDL may suppress the secretion of miR-29a and exert pro-atherosclerotic effects [108]. Decreased expression of miR-1246, miR-182, and miR-486 in SMC exosomes under the influence of PDGF promotes EC migration [109].

In addition to miRNAs, which are the main focus of this paper in EV, there are other substances (e.g., circRNAs [110] and heat shock proteins [111], etc.) that are also involved in EC–SMC communication and play distinct roles in AS formation.

#### 3.1.3. Extracellular Matrix Surrounding Endothelial Cells and Smooth Muscle Cells

The ECM is a complex network composed of collagen, non-collagenous proteins, elastin, proteoglycans, and glycosaminoglycans, etc. [112]. Both ECs and SMCs are capable of synthesizing and secreting ECM [113,114]. The vascular endothelium can form an endothelial glycocalyx, which blocks the adhesion of ECs to leukocytes. Under inflammatory stimulation, ECs and leukocytes can secrete related cytokines to remodel the glycocalyx barrier, thus promoting the interaction between ECs and leukocytes [115,116]. During the development of AS, SMCs migrate and produce ECM, forming a fibrous cap that covers the plaque surface [1], which can maintain the stability of the plaque.

ECM not only supports and connects cells but also plays an extremely important role in cell growth and proliferation as well as metabolism [112]. Firstly, the ECM can regulate cell activity through growth factors such as insulin-like growth factors and fibroblast growth factors (FGF) [117]. In most cases, the functions of these cytokines depend entirely on the adhesion of cells to the ECM. For instance, when ECs are prevented from adhering to the ECM, they can induce cell apoptosis and growth arrest [118]. Secondly, many physiological processes regulated by the ECM are achieved through the continuous remodeling of actin and the cytoskeleton [119]. Cadherins and integrins play important roles in it, primarily responsible for the mechanical integrity of tissues [120]. Cadherins mediate the connection between cells, which have been mentioned above. Whereas integrins mediate adhesion between cells and ECM [39], their signaling with other cell surface receptors such as growth factors, cytokines, and G-protein coupled receptors is also closely linked. Moreover, changes in the ECM can also alter the activity of different integrins [121], thereby regulating cell activities.

The components of the ECM, such as collagen, proteoglycans, and hyaluronic acid (HA), mediate or influence the communication between ECs and SMCs. Research has found that collagen V secreted by SMCs into the ECM may inhibit the recovery of adjacent endothelial damage caused by inflammation [122]. The ECM component Mindin/spondin 2 can prevent intimal thickening and vascular proliferation by inhibiting abnormal SMC proliferation, migration, and phenotypic transformation through an AKT-dependent manner [123]. Nevertheless, the expression of Mindin is significantly downregulated in SMCs and vascular tissues stimulated by PDGF-BB and guidewire injury, respectively. Furthermore, HA is highly present in the vascular media and new intima, facilitating SMC dedifferentiation, migration, and proliferation [124], thereby strongly contributing to vascular wall thickening. New research has further shown that treatment of SMC with PDGF-BB results in a decrease in the expression of proteoglycan link protein 1 (HAPLN1) [125]. Meanwhile, as a molecule that can assist in maintaining the stability of HA and proteoglycans in the ECM, exogenous HAPLN1 can significantly inhibit the proliferation, migration, and dedifferentiation of human aortic SMCs stimulated by PDGF-BB [126]. The balance and specific mechanisms of the effects of PDGF-BB and HAPLN1, as well as HA and HAPLN1 on SMCs, still need to be further elucidated.

The ECM can also mediate mechanical communication between ECs and SMCs, allowing the two cell types to communicate by responding to changes in the mechanical properties of the ECM induced by adjacent cells [127]. Abnormal mechanical communication can lead to the development of diseases such as AS [128]. The matrix protein thrombospondin-1 (Thbs1) acts as an extracellular mediator of matrix mechanical transmission and may protect cells under mechanical stress by increasing cell stiffness through the Thbs1/integrins/YAP signaling pathway. Simultaneously, inhibiting this pathway can reduce the proliferation of neointimal cells during carotid ligation [129,130].

### 3.2. Crosstalk Between Endothelial Cells and Macrophages

**Figure 3:**
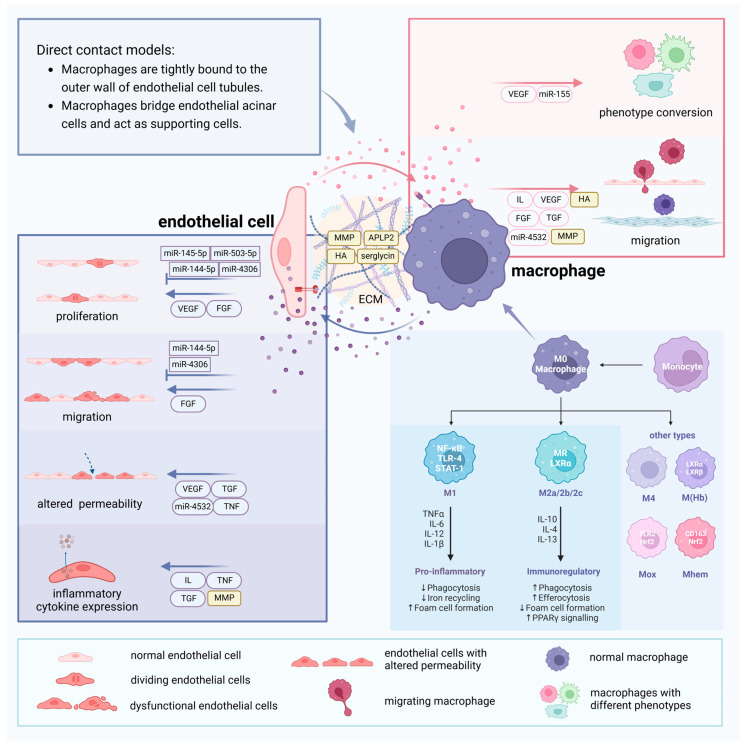
Crosstalk between endothelial cells and macrophages and different phenotypes of macrophages. Different modes of direct contact between endothelial cells and macrophages provide avenues for communication between these two cell types. Vasoactive substances as well as miRNAs contained within extracellular vesicles can lead to proliferation, dysfunction, changes in permeability, and expression of inflammatory factors in endothelial cells. Moreover, those can also induce phenotypic transformation and migration in macrophages. Additionally, components of the extracellular matrix can influence their communication. Macrophages are classified into two main phenotypes: M1, which secretes pro-inflammatory factors, and M2, which secretes anti-inflammatory cytokines. Beyond these, other phenotypes such as M(Hb), Mhem, HA-mac, Mox, and M4 also exist.

In AS, ECs primarily modulate the phenotypic conversion and migration of macrophages. Conversely, macrophages exert a reciprocal influence on ECs by either promoting or inhibiting their proliferation, migration, alterations in permeability, and production of inflammatory molecules (Figure 3).

#### 3.2.1. Direct Contact Between Endothelial Cells and Macrophages

There are two modes of direct contact between EC and macrophages [131]: macrophages are tightly bound to the outer wall of endothelial tubules; and macrophages bridge endothelial tip cells and act as supportive cells.

The conditioned medium of M1-type macrophages promotes angiogenesis, but when M1-type macrophages are directly cocultured with ECs, angiogenesis is inhibited [132], indicating that direct contact between ECs and macrophages suppresses vascular formation. Conversely, the Notch ligand Dll1 expressed by vascular ECs can mediate macrophage maturation and inhibit the proliferation of M1-type macrophages [133]. Still, more research is necessary to completely understand the precise chemicals behind these processes and how they affect vascular formation. ECs have the ability to successfully stimulate macrophage differentiation and M2-like polarization in vitro [134], and the close association between M2-type macrophages and ECs is conducive to promoting angiogenesis.

Macrophages can secrete VEGF-C to activate the Notch signaling pathway [135], thereby bridging endothelial tip cells at the post-vascular connections and providing the possibility for communication between ECs of different vascular segments [136]. Therefore, some scholars also believe that macrophages act mainly through molecular pathways rather than direct mechanical means [135].

#### 3.2.2. Paracrine Secretion Between Endothelial Cells and Macrophages

##### Vasoactive Substance Between Endothelial Cells and Macrophages

Substances that mediate intercellular communication between the two types of cells, EC and macrophages, include VEGF [137], IL [138], TGF [139], TNF [140], and FGF [141].

VEGF is now believed to stimulate EC proliferation, increase vascular permeability, and induce macrophage migration [45]. Within the hemorrhagic regions of plaques, M(Hb) macrophages can enhance vascular endothelial permeability and promote plaque inflammation by promoting the expression of VCAM on plaque endothelium through VEGF-A [137]. Additionally, both in vitro [142] and in vivo [143] experiments have proven that macrophages expressing stable overexpression of VEGF are capable of transdifferentiating into endothelial-like cells. These cells can impede the advancement of AS by mending early-damaged artery ECs and encouraging repair through the release of VEGF and localized NO production. This finding provides insights for future macrophage-targeted therapies against AS. Furthermore, a study using ECs specifically deficient in the glycolytic regulator phosphofructokinase-2/fructose-2,6-bisphosphatase isoform 3 (PFKFB3) revealed that ECs can secrete lactic acid to signal macrophages toward an M2-like phenotype polarization through a mechanism dependent on monocarboxylate transporter 1 s(MCT1) [144], thereby promoting VEGF secretion and angiogenesis. Subsequently, VEGF derived from macrophages reinforces this circuit, leading to the activation of angiogenic potential in microvascular endothelium.

IL was originally produced by leukocytes and functioned as a cytokine between leukocytes, all of which are small molecule peptides or glycoproteins [145]. In the IL family, IL-1 is a significant factor that mediates inflammation and can be produced by both ECs and macrophages. IL-1 can promote the production of IL-8 by ECs [138], which may recruit monocytes and other cells to the site of inflammation, and thus promoting the formation of new blood vessels [146]. In the early stages of AS, the subtype IL-1β secreted by macrophages can induce inflammation in ECs, such as increasing the expression of adhesion factors and chemokines [147]. IL-1β–induced inflammatory molecules in ECs can further enhance macrophage activity, and induce the onset of inflammatory responses [148].

TGF is a peptide that regulates cell growth and differentiation, primarily produced by macrophages. M1-type macrophage-derived foam cells can induce endothelial-to-mesenchymal transition (EndMT) by upregulating chemokine ligand 4 (CCL-4, a member of the TGF family), increasing endothelial permeability, and promoting monocyte adhesion [139]. Inhibiting the TGF-β signaling pathway in ECs can reduce the expression of VCAM-1 and ICAM-1 on ECs and the recruitment of leukocytes, thereby inhibiting the progression of AS [149]. Some studies have also suggested that TGF-β can carry out pro-angiogenic and anti-angiogenic effects through different pathways, which may have different effects on EC proliferation and migration, etc.

TNF is a small molecule protein that drives inflammatory responses and is typically produced in individuals with inflammation or disease. Specifically, TNF-α, predominantly secreted by macrophages, exerts significant influence on the function of ECs. Upon stimulation by tissue injury or inflammatory cytokines such as interferon(IFN)-γ, TNF-α secreted by macrophages promotes the expression of CAMs on the surface of vascular ECs, including E-selectin, VCAM-1, and ICAM-1 [140]. It can also induce endothelial dysfunction associated with increased oxidative stress and decreased expression of eNOS protein [150,151]. Furthermore, TNF-α disrupts the stability of EC cytoskeleton, triggering cytoskeletal rearrangements [152] that lead to functional impairment of the ECs.

FGF is a polypeptide of about 150–200 amino acids, with at least 20 members in the family. The main member involved in atherosclerotic lesions is the basic fibroblast growth factor (bFGF). bFGF is secreted by SMCs and macrophages, which exerts its activity by binding to different types of cell surface FGF receptors (FGFRs) on cells, including ECs and SMCs. This process produces signals that promote cell motility, proliferation, and survival, leading to angiogenesis. Activated endothelial FGF-R2 signaling promotes the synthesis of adhesion molecules in ECs, facilitating the migration of inflammatory cells to the subendothelial space to promote the development of AS [141]. Additionally, in vivo studies [153] have demonstrated that by inhibiting FGFR1 phosphorylation and blocking bFGF signaling, the migration, proliferation, and angiogenesis of ECs can be reduced. This ultimately decreases intralesional angiogenesis and plaque hemorrhage.

##### Extracellular Vesicle Between Endothelial Cells and Macrophages

It has been proposed that ox-LDL–stimulated macrophages can inhibit EC growth by secreting exosomes [154]. New researches further reveal that macrophage-derived EVs carrying miR-144-5p [155], miR-145-5p [156], miR-223 [157], miR-503-5p [158], miR-199a-5p [159], miR-4532 [160], miR-4306 [161], and other miRNAs can affect EC. A study using miR-144-5p mimics for in vitro experiments surmised that macrophage-derived miR-144-5p can inhibit EC proliferation and migration, and induce apoptosis [155]. Under this, miR-145-5p may suppress LPS-induced EC proliferation by regulating macrophage polarization to the M2 phenotype [156]. Microvesicles released by activated human macrophages were able to transport miR-223 to ECs to exert its effects [157]. Macrophages can also secrete exosomes containing miR-503-5p to inhibit human coronary artery EC proliferation and angiogenic function and promote AS formation [158]. Under the stimulation of ox-LDL, the expression of miR-199a-5p in EVs derived from macrophages is downregulated, which promotes EC pyroptosis via the SMARCA4/PODXL/NF-κB axis and thereby accelerates AS [159]. Furthermore, macrophage-derived exosomes carrying miR-4532 can cause EC damage by targeting SP1 and activating downstream NF-κB P65 in response to ox-LDL stimulation, while positive feedback increased the attraction to macrophages, exacerbating foam cell formation and the transfer of exosomal miR-4532 to ECs [160] and promoting AS formation. ox-LDL can also stimulate macrophages to release EVs containing miR-4306 to inhibit proliferation and migration and angiogenesis of EC from coronary vessels in vitro [161]. Further studies on how macrophages balance the release of these three miRNAs to regulate the course of AS are warranted.

Correspondingly, EC-derived EVs carrying miR-10a [162], miR-155 [163], and other miRNAs can also affect macrophage function. Resting healthy ECs can secrete EVs carrying miR-10a to transfer to monocytes, and target factors such as IRAK4, β-TRC, and MAP3K7 in the NF-κB pathway to inhibit inflammatory signaling [162]. Ox-LDL stimulation can induce ECs to produce EVs rich in miR-155 to promote monocyte activation and shift monocytes/macrophages from anti-inflammatory M2 to pro-inflammatory M1 phenotypes. EVs secreted by human coronary artery ECs expressing Krüppel-like factor 2 can inhibit inflammation and monocyte activation, and AS formation [163]. So different exosomes may have different pro-inflammatory and anti-inflammatory effects.

#### 3.2.3. Extracellular Matrix Surrounding Endothelial Cells and Macrophages

Macrophages can secrete substances that degrade ECM proteins, such as matrix metalloproteinases (MMPs). MMPs are a family of zinc-dependent endopeptidases [164], and their production is closely related to the rupture of the fibrous cap in atherosclerotic plaque. Proteinase-activated receptors (PARs) are important targets for MMPs. Activation of PAR-2 stimulates endothelial NO production and promotes vasodilation via the Ser1177-eNOS phosphorylation pathway [165]. EC-produced MMP-10 can regulate macrophage migration and invasion [166]. Most studies have focused on the changes in MMP expression in plaques and their role as biomarkers, with few studies reporting on MMP-mediated cell communication and its specific mechanisms. On the other hand, macrophages can also synthesize molecules that contribute to the formation and stability of the ECM, such as the proteoglycan APLP2 and serglycin [167]. These molecules play a role in the ECM changes that occur during AS development. However, how they contribute to the development of AS, and whether they aid in pro- or anti-inflammatory processes, remains to be determined.

Moreover, chemokines on ECs and their secreted ECM components primarily promote the adhesion and transfer of monocytes. For example, HA in the ECM produced by ECs can mediate macrophage translocation through the HA receptor Lyve-1, indicating their position through the EC gap [124].

### 3.3. Crosstalk Between Smooth Muscle Cells and Macrophages

**Figure 4:**
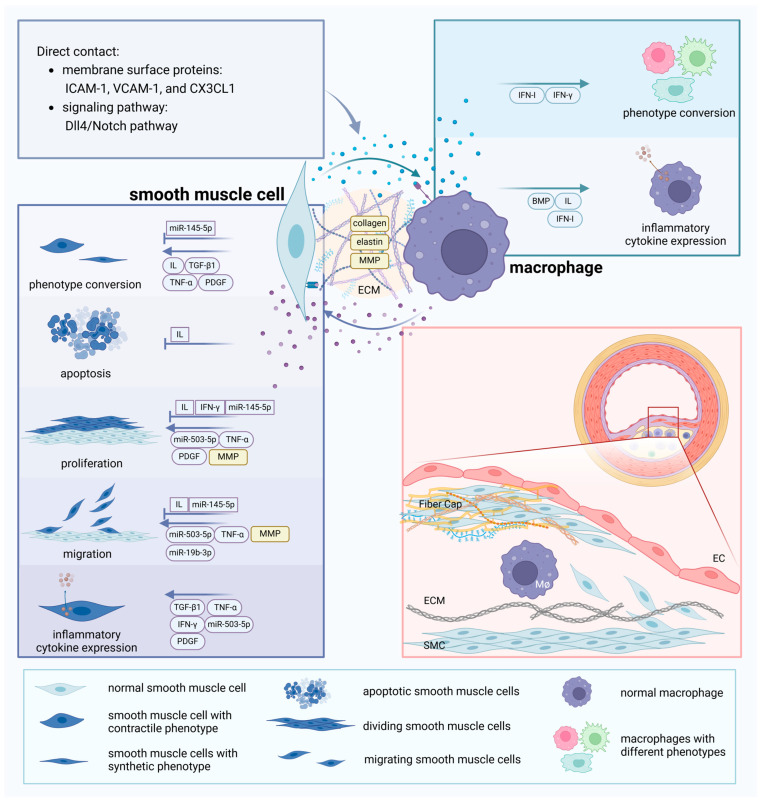
Crosstalk between smooth muscle cells and macrophages and atherosclerotic scenarios in the vasculature. Membrane surface proteins like ICAM-1, VCAM-1, and CX3CL1 can mediate the interaction between smooth muscle cells and macrophages, or they can interact directly through signaling pathways like Dll4/Notch. Vasoactive substances such as IL, TGF, TNF, IFN, and PDGF play crucial roles in the crosstalk between smooth muscle cells and macrophages. Factors secreted by both types of cells can influence smooth muscle cell phenotypic switching, apoptosis, proliferation, migration, and changes in inflammatory factor expression, as well as affect macrophage phenotypic conversion and the expression of inflammatory factors in atherosclerosis. miRNAs such as miR-503-5p and miR-145-5p in extracellular vesicles also play a significant role in this process. The extracellular matrix, which constitutes the cellular environment, can be altered by the communication between macrophages and smooth muscle cells.

The secreted factors from SMCs and macrophages engage in a complex interplay that modulates disease progression through both pro-atherogenic and anti-atherogenic mechanisms. This interaction influences SMC apoptosis, proliferation, and migration, while also affecting phenotypic conversion and the secretion of inflammatory cytokines in both cell types(Figure 4).

#### 3.3.1. Direct Contact Between Smooth Muscle Cells and Macrophages

Evidence indicates that two cell types may engage in interactions mediated by membrane surface proteins including ICAM-1, VCAM-1, and CX3CL1 [168]. ICAM-1 and VCAM-1 are largely expressed on ECs and macrophages, with a detectable presence on SMCs, where they significantly contribute to the adhesion and recruitment of macrophages. Hashem et al. used mesenchymal stem cell therapies to replace SMCs in the neointima, reducing the expression levels of adhesion molecules and markers of oxidative and inflammatory stress, such as COX-2, iNOS, and TNF-α [169]. This reduction subsequently inhibited the migration of monocytes and the formation of foam cells by macrophages, underscoring the intricate relationship between macrophages and SMCs in the AS process. Furthermore, the engagement between SMCs and monocyte/macrophages can also augment the expression of TNF-α, IL-1β, IL-6, and MMP through the CX3CL1/CX3CR1 pathway [170], suggesting a potential mechanism by which these cells contribute to the promotion of AS.

Furthermore, a study has shown that in a direct coculture system of SMCs and macrophages, M1-type macrophages can facilitate the transition of SMCs from a contractile to a synthetic phenotype via the Dll4/Notch pathway [171]. This transition is known to enhance the migration of SMCs and promote the progression of AS. Moreover, macrophages can induce apoptosis in SMCs within plaques through direct contact, a process influenced by Fas/Fas-L interaction [172] and NO [173].

Consequently, researches on the direct contact between SMCs and macrophages mainly occur in vivo or in direct coculture systems, regulating the inflammatory process of AS through various pathways. However, scholars including Pinhao Xiang [10] have found that in human AS lesions, SMCs and macrophages are relatively segregated and maintain distinct positions. In contrast, in mouse models of the disease, SMCs migrate to the intima only after the induction of AS and rapidly mix with macrophages. This suggests that findings from different disease models may vary, indicating a need for further exploration in this field.

#### 3.3.2. Paracrine Secretion Between Smooth Muscle Cells and Macrophages

##### Vasoactive Substance Between Smooth Muscle Cells and Macrophages

ILs [174], TGF [175], TNF [176], IFN [177], and PDGF [178] are all important factors that mediate communication between macrophages and SMCs.

Macrophages are capable of secreting ILs that induce metabolic and morphological changes in SMCs. In co-culture systems of macrophages and SMCs, IL-5 overexpressing macrophages have been shown to inhibit Ang II–induced apoptosis in SMCs [174]. IL-10 secreted by macrophages can bind to IL-10 receptors on rat vascular SMCs, directly inhibiting SMC proliferation and migration [179]. Studies have linked coronary artery calcification to increased levels of IL-18. IL-18 can activate SMCs through the transient receptor potential melastatin 7 (TRPM7) channel, which is a Mg^2+^ and Ca^2+^ permeable ion channel, thereby driving SMC differentiation into an osteoblast-like phenotype and promoting vascular calcification [180]. SMCs also secrete ILs to communicate with macrophages; for instance, in diseases such as allergic asthma, IL-11 secreted by SMCs can inhibit macrophage activity [181]. Additionally, treatment with an anti-IL11 antibody in SMCs has been associated with decreased levels of common macrophage markers such as galectin-3 and protein-2 [175]. However, research on the role of these interactions in AS is currently limited.

TGF-β1 produced by macrophages can promote SMC secretion of IL11, which in turn induces an extracellular regulated protein kinase (ERK)–dependent shift of SMC to the synthetic phenotype [175]. It also stimulates ECM expression and exerts an anti-inflammatory effect [182]. TGF-β1 can inhibit SMC activation and the inflammatory response in SMCs by mediating the Smad3 pathway [183]. Moreover, bone morphogenetic protein (BMP), a member of the TGF-β family, is produced by SMCs and has been shown to be closely related to macrophages recruitment. In early AS formation in ApoE-/- mice, the expression of BMP-2 and BMP-4 genes in SMCs is upregulated, promoting monocyte recruitment and inflammation [184]. This highlights the dual nature of TGF-β, and how it balances pro-inflammatory and anti-inflammatory effects during AS development still remains to be investigated.

In AS, TNF-α secreted by macrophages can induce proliferation, migration, and synthetic behavior in SMCs [176]. It induces the differentiation of SMCs into osteoblast-like cells, leading to calcium deposition and arterial sclerosis [185]. TNF-α also upregulates the activity of NOX enzyme complex in human aortic SMCs, contributing to the generation of oxidative stress and vascular dysfunction [186]. Consequently, TNF-α plays an important role in promoting the progression of AS.

IFNs were initially identified as protein substances that interfere with viral replication and involved in physiological activities such as cell proliferation and immune response. They are primarily classified into three types: Type I, Type II, and Type III, with Type II IFN consisting only of the IFN-γ subtype. IFN-γ inhibits the proliferation of rat SMC by inducing the production of NO by NO synthase [187]. Meanwhile, IFN-γ stimulates the differentiation of monocytes to macrophages and promotes macrophage activation and foam cell formation [177]. Long-term in vivo studies have suggested that IFN-I can promote the recruitment of monocytes, and these monocytes/macrophages may mature into SMC-like macrophages or macrophage-like SMCs [188], thereby contributing to the development of AS. However, it has also been shown that IFN-β, an IFN-I isoform, attenuates Ang II–induced AS in ApoE−/− mice and exerts anti-inflammatory effects [189]. This may be associated with different downstream signaling pathways of IFN-I, such as the NF-κB pathway, the PI3K/AKT pathway, and the MAPK pathway.

Increased PDGF receptor β signaling in SMCs promotes the production of chemotactic factors [190], which induce SMC dedifferentiation and proliferation, ECM secretion, and monocyte recruitment. This not only facilitates the formation of advanced plaque morphology but also promotes the formation of new plaques in the thoracic aorta. Correspondingly, in SMCs in PDGFR-β knockout mice with a long-term Western diet, there is a decrease in collagen content in the fibrous cap and an increase in intraplaque hemorrhage [178]. This means that long-term absence of PDGF receptor β in SMCs leads to reduced stability of the fibrous cap and plaques.

##### Extracellular Vesicle Between Smooth Muscle Cells and Macrophages

Macrophages deliver miR-503-5p to human coronary SMC via EV, which downregulates Smad7, smurf1, and smurf2 and elevates TGF-β1 to promote SMC proliferation and migration and exacerbate AS [158]. EVs containing miRNA-19b-3p that originate from macrophages promote the migration of VSMCs and the progression of AS by targeting JAZF1 [191]. In addition, overexpression of miR-145-5p inhibits PDGF-induced proliferation, migration, and phenotypic transformation of VSMCs [192], closely associated with the Smad4 and TGF-β signaling cascade. In contrast, EVs produced by macrophages treated with LPS can induce inflammation and oxidative stress in SMCs, exacerbating the process of vascular calcification [193]. Most studies have focused on in vitro experiments [194], and more research is needed to combine in vitro and in vivo experiments to clarify whether the role of miRNAs in vivo differs from that observed in vitro, thus holding promise for the diagnosis and treatment of diseases.

#### 3.3.3. Extracellular Matrix Surrounding Smooth Muscle Cells and Macrophages

Communication between macrophages and SMCs may alter the composition of ECM and the expression of factors related to neoangiogenesis. For instance, in macrophage and SMC co-cultures, the expression of ECM proteins, such as collagen I, collagen III, and elastin, is decreased in SMC, while the expression and activity of MMP-9 and MMP-1 are increased [195].

SMCs not only secrete components of the ECM but also release various MMPs that can digest ECM proteins. MMP-induced degradation of ECM proteins may lead to plaque instability [196]; however, the ability of MMPs to promote SMC migration and proliferation may contribute to the growth and stability of atherosclerotic plaque caps [197]. Thus, MMPs exhibit a dual role in this process, yet most scholars still believe that MMP-induced destruction of the ECM weakens the protective fibrous cap.

Peripheral blood monocytes and macrophages constitutively secrete MMP-9, which is crucial for SMC proliferation and migration to the endothelium, as well as its involvement in the reorganization of the collagen matrix [198]. It significantly impacts the progression of AS and vascular restenosis. In addition to MMP-induced degradation of ECM proteins and thinning of the fibrous cap, macrophage secretions (e.g., TGF-β) also lead to a reduction in collagen synthesis by SMCs [199], thereby thinning the fibrous cap and destabilizing the plaque.

It is clear that the ECM is the site of cellular survival and establishes the conditions for cellular activity and, in turn, cells actively remodel their surrounding ECM to maintain microenvironment stability.

### 3.4. Crosstalk Among All Three Cell Types

During the progression of AS, the interactions between ECs, SMCs, and macrophages are not isolated but rather form an interdependent and complex network. Each cell type within this network plays multiple roles, functioning as both initiators and recipients of signaling, as well as modulators. Obviously, in some instances, all three cell types participate in specific crosstalk mechanisms, further underscoring their synergistic roles in the pathological progression of AS.

ECs and SMCs can influence the polarization and function of macrophages by secreting signaling molecules such as chemokines and cytokines. Conversely, macrophages also exert reciprocal effects on ECs and SMCs. For instance, when ECs are subjected to oxidative stress or inflammatory stimuli, they release monocyte chemoattractant protein-1 (MCP-1) and other factors, recruiting monocytes to the vascular wall and promoting their differentiation into macrophages [200]. These macrophages then continuously phagocytose ox-LDL. Upon stimulation by ox-LDL [201] and LPS [140], macrophages secrete TNF-α, which increases the expression of adhesion molecules and chemokines on the surface of ECs [140]. This process recruits additional monocytes into the arterial wall, where they differentiate into macrophages. Additionally, studies have shown that in high-fat diet-fed ApoE−/− mice and in patients with AS, TNF-α activates the NF-κB signaling pathway, promoting the generation of miR-155. This leads to the transition of VSMCs from a contractile to a synthetic phenotype and promotes their proliferation and migration [202]. Clonally expanding VSMCs promote inflammation by upregulating Complement Component 3 (C3), while simultaneously evading macrophage clearance by upregulating Cluster of Differentiation 47 (CD47), leading to an increase in plaque volume [203]. In the late stages of AS, increased TNF-α expression in neointimal SMCs stimulated by IL-1β and IFN-γ sensitizes these cells to apoptosis, thereby contributing to plaque rupture [204].

This network encompasses not only paracrine signaling between cells but also involves significant contributions from EV-mediated communication. Macrophages can secrete EVs that deliver miR-503-5p to human coronary artery ECs and SMCs. This transfer of miRNA not only inhibits the proliferative and angiogenic functions of ECs but also constrains the proliferative and migratory capacities of SMCs [158]. Antagonistic miR-33a-5p expressed by ECs is transferred to macrophages and SMCs via EVs, promoting cholesterol efflux in both cell types. Consequently, it is hypothesized that EC-derived miR-33 may promote lipid accumulation in ECs and, through exosome-mediated transfer, enhance the endogenous effects of miR-33a-5p. This process inhibits cholesterol efflux in macrophages and SMCs, thereby facilitating the development of AS [205].

Several studies have constructed in vitro three-cell co-culture models of EC, macrophages, and SMCs to better investigate the crosstalk among these three cell types, with different models focusing on different aspects [206]. Aspects including lipid accumulation [207], macrophage migration and foam cell formation [208], and immune–vascular interactions [209] are the subject of many models. A recent study introduced a new human AS three-cell direct co-culture model, which mimics the early to mid-stage of the natural history of AS [210]. This model has low shear stress and LDL conditions that are comparable to those in human AS. Meanwhile, the model’s construction took only 24 h, greatly improving experimental efficiency and offering a valuable method for researching the pathogenesis of AS. We may keep investigating the communication between three cells based on this remarkable three-cell co-culture method.

## 4. Conclusions and Perspective

The communication between ECs, SMCs, macrophages, and related molecules—which either stimulate or inhibit AS through various pathways—under the effect of ox-LDL, shear stress, free radicals, and mechanical stress is summarized in this review. This intricate intercellular crosstalk involves a variety of key molecules, particularly cytokines, growth factors, and other vasoactive substances, as well as miRNAs encapsulated within EVs. Vasoactive substances such as Ang II and ET exacerbate vascular inflammatory responses and promote plaque formation by inducing the contraction and proliferation of VSMCs. Meanwhile, molecules such as TNF-α and IL-1 induce EC dysfunction and pro-inflammatory responses, facilitate the phenotypic switching of VSMCs or the migration of macrophages. miRNAs within EVs, such as miR-155, promote the polarization of macrophages toward a pro-inflammatory phenotype, further destabilizing the plaque. Conversely, NO, CO, and PGI_2_ contribute to vascular homeostasis by promoting vasodilation and SMC relaxation. Additionally, miRNAs such as miR-143/145, miR-4532, and miR-144-5p enhance plaque stability by regulating SMC phenotypic switching and suppressing EC dysfunction.

Furthermore, certain molecules exhibit dual regulatory roles in the progression of AS. For instance, VEGF and PDGF can accelerate plaque progression by promoting angiogenesis and cell proliferation, yet they may also exert protective effects during vascular repair. Similarly, TGF-β can exacerbate plaque inflammation through pro-inflammatory actions, while its anti-inflammatory effects can promote plaque stability. The diverse effects of these molecules on cells may depend on the stimulatory context and cell type. For example, TGF-β strongly inhibits the proliferation and migration of ECs, which is associated with increased synthesis of ECM and proteoglycans [211]. In SMCs, TGF-β signaling, activated by inhibiting FGF signaling, promotes the phenotypic switch of SMCs from a synthetic to a contractile phenotype, thereby reducing SMC numbers in the plaque and limiting plaque growth [212]. Conversely, blocking FGF signaling in ECs can activate TGF-β signaling, inducing EndMT, which leads to an increase in the number of SMCs and fibroblasts, as well as enhanced ECM deposition [213]. On the other hand, activation of FGF signaling in ECs recruits immune cells, such as monocytes/macrophages, to the subendothelial space, thereby promoting AS [141].

Although the involvement of different molecules in cellular communication and their impact on the development of AS have been understood, the specific mechanisms by which these molecules function in AS are still not fully elucidated. Meanwhile, a variety of molecules have distinct functions during different phases of the illness, which could be connected to the disease’s fluctuating cellular makeup. The exact reasons for this phenomenon remain to be explored. We should not limit our attention to a single cell type or substance because the progression of AS always involves numerous cell types and is accompanied by complicated molecular signaling. Rather, we ought to look into the interactions between cells and the influence of different factors on each other. In AS, the substances secreted by cells are always under dynamic change, and the imbalance between pro-inflammatory and anti-inflammatory substances may lead to changes in the direction of AS development. This could provide a new perspective for studying the causes of AS as well as its treatment.

Currently, more research has been carried out on the interactions between ECs and SMCs, but less has been explored on the crosstalk between the two cells and macrophages [214], particularly when it comes to direct contact and the ECM. Not only may EC, SMC, and macrophages communicate with each other to affect each other’s growth and metabolism, but they can also use autocrine regulation to alter the behavior of the same cells [215,216]. But few studies have clearly defined the conditions in which these cells must communicate, or under what circumstances both autocrine and communication are required to fulfil normal physiological activities. Investigations into these issues are still pending.

These three cells are not the only immune cells that impact the AS process; T cells, B cells, neutrophils, and other cells also release various cytokines that impact the course of AS. These cells may also exhibit different forms of communication and crosstalk with each other. It would be worthwhile to explore whether analyzing the communication of other cell types can lead to a more profound understanding of AS progression.

In addition, subtle differences in the selection of experimental models may contribute to the significant disparities between in vitro experimental results and clinical trial outcomes. Factors such as racial differences (between humans and mice) mentioned above, as well as gender differences and variations in blood vessel types, can influence the results. According to recent research, the Y chromosome in humans has an impact on the immune system and inflammatory responses. This leads to the fact that there is a more pronounced pro-inflammatory gene expression in male M1 macrophages during LDL internalization, while female M1 macrophages secrete more substances associated with cell damage [217]. Moreover, a study compared the commonly used model in experiments with the actual human atherosclerotic process for the first time. They found that the histological features of the different stages of AS in human coronary arteries and aorta were similar, but there was no intraplaque hemorrhage in aortic lesions [218]. All these differences need to be carefully considered and addressed in future experiments to provide references for clinical treatment.

Researching the communication mechanism can yield more insights into potential future therapies for AS disorders. For instance, blocking intercellular communication or modifying the targeting of intercellular communicating substances could be therapeutic approaches. As previously noted, miRNAs contained in EVs play an important role in intercellular communication. A recent study has discovered that a unique sequence on miRNAs in cells, called motif, controls whether or not those miRNAs are loaded into vesicles and passed on to other cells [219]. Recoding the motif of cells through RNA editing techniques may represent a viable option for AS treatment.

In summary, communication between ECs, SMCs, and macrophages plays a crucial role in the progression of AS. Human intervention in their communication process can offer insights into potential therapeutic approaches for the disease in the future.
